# How sports clubs decide to adopt an outdoor smoke-free policy: a qualitative study applying the Garbage Can Model

**DOI:** 10.1186/s13011-022-00487-x

**Published:** 2022-07-20

**Authors:** Heike H. Garritsen, Andrea D. Rozema, Ien A. M. van de Goor, Anton E. Kunst

**Affiliations:** 1 Department of Public and Occupational Health, UMC Location University of Amsterdam, Amsterdam, The Netherlands; 2 Tranzo Scientific Center for Care and Wellbeing, Tilburg School of Social and Behavioral Sciences, Tilburg University, Tilburg, The Netherlands

**Keywords:** Smoke-free policy, Decision-making, Sports clubs, Garbage can model

## Abstract

**Background:**

Outdoor smoke-free policies (SFPs) at sports clubs can contribute to protecting people from second-hand smoke (SHS). However, in absence of national legislation, it is uncertain whether and how sports clubs decide to adopt an SFP. The aim of this study was to explore the decision-making process at sports clubs in relation to the adoption of an outdoor SFP.

**Methods:**

Semi-structured interviews were held with key stakeholders at 20 Dutch sports clubs (in field hockey, football, tennis, or korfball) with an outdoor SFP. Thematic analysis was applied, and themes were defined in line with the four streams of the Garbage Can Model (GCM).

**Results:**

We identified four motivating factors for sports clubs to start the decision-making process: 1) SHS as a problem, 2) intolerance of smoking behavior, 3) advantages of an outdoor SFP, and 4) external pressure to become smoke-free. The decision-making process involved a variety of participants, but the board, influential club members, and smokers usually played major roles. Decisions were discussed during both formal and informal choice opportunities, but only made during formal choice opportunities. With regard to solutions, sports clubs adopted a partial or total outdoor SFP. In addition, sports clubs followed different strategies with regard to the decision-making process, which we classified along two dimensions: 1) autocratic vs. democratic and 2) fast vs. slow.

**Conclusion:**

A number of factors motivated sports clubs to start the decision-making process. These factors were mainly linked to a strong non-smoking norm. Decision-making involved different participants, with a key role for the board, influential club members, and smokers. Governments and other external organizations may contribute to SFP adoption at sports clubs in several ways. They may advise clubs on strategies of decision-making and how to involve smokers in this process.

## Background

Negative health effects associated with exposure to secondhand smoke (SHS) have been well documented and include heart disease, respiratory disease, and cancer [1]. The most effective approach to reduce SHS exposure among non-smokers is the implementation of policies that establish smoke-free environments [2]. Such smoke-free policies (SFPs) not only decrease SHS, but also the social acceptability and visibility of smoking [3] and may encourage smokers to reduce their tobacco use or quit smoking completely [4, 5].

Initially, SFPs focused on banning smoking in indoor public places, such as workplaces, restaurants, and bars [6]. More recently, countries have also implemented policies that prohibit smoking in outdoor settings, such as in parks or at sporting venues [7]. The latter has significant potential to protect people from SHS as sports plays an important role in the life of many people [8]. Sports clubs are an important arena in which to perform sport. For example, in Europe, there are about 700,000 sports clubs with an estimated 70 million members [9]. Nevertheless, in many countries, sports clubs remain one of the public places where smoking is currently not banned.

In the absence of legislation and regulation, some sports clubs in various countries (e.g., in Australia, Ireland, and the Netherlands) have voluntarily adopted an outdoor SFP [10, 11]. Yet, many clubs refrain from adoption. In a previous study, we found that although sports clubs recognize the intrinsic value of an outdoor SFP, they foresee a number of practical issues (e.g., losing smoking volunteers, expected problems with enforcement) that may inhibit them from adopting an outdoor SFP [12]. To increase the number of sports clubs with an outdoor SFP, it is important to develop a better understanding of why some clubs do adopt such policies while others do not.

Nonprofit organizations like sports clubs have a specific mode of decision-making that can be represented by the Garbage Can Model (GCM) [13]. According to the GCM, decision-making processes do not follow linear sequences of decision-making steps. Instead, the outcome of the decision process stems from four concurrent streams, defined in terms of problems, participants, choice opportunities, and solutions. Problems are the issues that cause concern and may be raised by people within or outside the organization. Participants are the individuals who raise the problem, discuss the options and make a decision. Choice opportunities are occasions in which decisions are sought, discussed, and made. Finally, solutions present the range of possible responses to the identified problems to be decided upon. Frequently, solutions arise before the problems, as initiatives that are on the lookout for corresponding needs [13].

Schlesinger et al. [14] found the GCM to be a suitable model with which to interpret the decision-making at sports clubs. Using the model, they examined how sports clubs recruit volunteers by analyzing the decision-making processes underlying their practices. They showed that decisions often resulted from a response to acute recruiting problems rather than a pursuit of strategic goals. Furthermore, decisions took a relatively superficial course, with a limited amount of time spent on discussions along with the limited depth of these discussions [14]. Until now, no study has addressed decision-making processes at sports clubs in relation to the adoption of an outdoor SFP.

The current study aimed to explore the decision-making process at sports clubs that did adopt an outdoor SFP. More specifically, we explored key stakeholders’ perceptions regarding the decision-making process. The GCM served as a guidance for developing the interview guide, conducting the analysis, and reporting the results.

## Methods

### Study design

This study used a qualitative design. Semi-structured interviews were held with key stakeholders at 20 sports clubs to identify and elucidate the decision-making process in relation to the adoption of an outdoor SFP.

### Research context: sports clubs in the Netherlands

Sports clubs in the Netherlands have several features that often apply to sports clubs in other countries as well [15–17]. First, they are autonomous organizations, i.e., they can act independently of public authorities and can decide their own policies. Second, sports clubs are non-profit associations. Their primary goal is not to maximize profits but to meet the needs of the club members. Third, most sports clubs are primarily run by volunteers, which are often members of the sports club. Finally, sports clubs have a democratic decision-making structure. Club members are expected to decide together on the sports club’s policy, while the board is held accountable for their actions during the (yearly) general membership meeting [15–17].

### Participants

Recruitment of the sports clubs was conducted by the first author in collaboration with three Public Health Services. We aimed to only include sports clubs that had become smoke-free in 2020 or 2021, so as to make sure that interviewees could remember the decision-making process. In order to increase the total number of clubs to a minimum of 20 we also included three clubs from 2019 and one from 2018. Documentation on the year of implementation of the outdoor SFP was provided by the Dutch Heart Foundation, which keeps track of smoke-free sports clubs in the Netherlands [11]. Board members of sports clubs were contacted by email, phone, or letter, and asked whether a member of the club wanted to share his/her perspectives regarding the decision to adopt an outdoor SFP. To represent the diversity of outdoor sports in the Netherlands, football, korfball, field hockey, and tennis clubs were included in the study. Participating clubs did not only vary in type of sports, but also in size (number of members) and degree of urbanization. The latter was based on the address density of the municipality in which the sports club was located and ranged from not urbanized (< 500 addresses per km2) to most urbanized (> 2500 addresses per km2) [18].

Semi-structured interviews were held with key stakeholders (*n* = 20, one stakeholder per club), i.e., club members who were involved in the decision-making process.

### Procedure

The study was approved by the Medical Ethics Review Committee of the Academic Medical Center (letter W20_318 # 20.369) and informed consent was obtained from all respondents included in the study. Interviews were conducted by the first author between March and September 2021. Due to COVID-19 restrictions, all interviews took place online via Microsoft Teams. All respondents gave permission to audio recordings. Interviews lasted on average 25 min. Respondents received a €35 gift card for their participation.

### Measures

The development of the topic guide was inspired by the four streams of the GCM [13]. For each stream (problems, participants, choice opportunities, solutions), a number of questions were formulated. Examples of questions during the interviews were: “Which people within the sports club were involved in the decision-making process?” and “When were discussions about the outdoor smoke-free policy held?”. The topic guide can be found in 36.

After the interview, respondents were sent a short questionnaire via email. This contained questions about their demographics (gender, age, educational level, and function within the sports clubs) as well as questions about the sports clubs (size of the sports clubs and year of implementation of the outdoor SFP). Characteristics of the participating sports clubs are presented in Table 1.

### Analysis

Interviews were transcribed verbatim by an external transcription company. Thematic analysis, a method for identifying themes within qualitative data, was applied, following an approach described elsewhere [19]. Within this thematic analysis, we incorporated both an inductive and a deductive approach. Using an inductive approach, we coded themes that emerged directly from the data. Next, the GCM was applied as a means of organizing the themes. Coding was conducted by the first author using MAXQDA [20] and another researcher randomly coded seven transcripts (35%) in parallel. Inconsistencies regarding codes were discussed until consensus was reached. Next, similar codes were pooled, resulting codes were rearranged, and themes were defined in line with the four streams of the GCM. The appropriateness of the developed themes, in relation to both the code list and theoretical model, was discussed with all authors and amended when necessary. With regard to the streams of the GCM, we decided to refer to ‘motivating factors’ instead of ‘problems’ since we believed that the latter term did not cover all sports clubs’ reasons to start the decision-making process.

## Results

Table 1 presents the characteristics of the participating sports clubs. Respondents were equally distributed by gender and their age ranged from 25 to 67 years. Most respondents (*n* = 19, 95.0%) were non-smokers. Furthermore, respondents varied in their function within the sports clubs, with the majority of respondents being a board member or committee member.

**Table 1:** Characteristics of the participating sports clubs

	**No. of sports clubs**	
	***n* = 20**	**%**
**Type of sports**		
Football	5	25.0
Korfball	5	25.0
Field hockey	5	25.0
Tennis	5	25.0
**Size of the sports clubs**		
< 250 members	5	25.0
250–500 members	4	20.0
500–1000 members	2	10.0
1000–1500 members	9	45.0
**Degree of urbanization (no of addresses/km2)**		
Most urbanized (> 2500)	8	40.0
Strongly urbanized (1500–2500)	3	15.0
Moderately urbanized (1000–1500)	1	5.0
Hardly urbanized (500–1000)	6	30.0
Not urbanized (< 500)	2	10.0
**Year of implementation of outdoor SFP**		
2018	1	5.0
2019	3	15.0
2020	13	65.0
2021	3	15.0

**Fig. 1:**
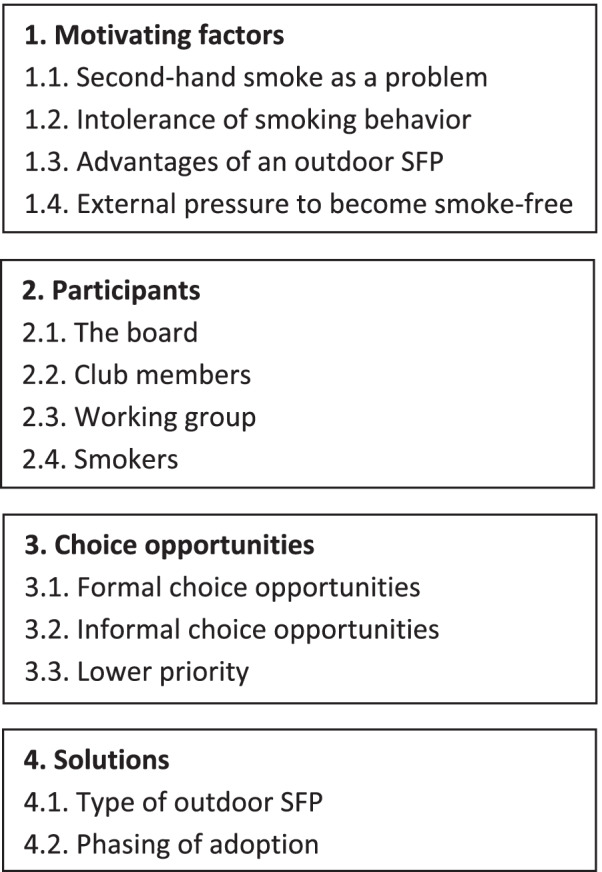
The defined themes in line with the four streams of the GCM

In this section, we first describe the results according to the four streams of the GCM. Thereafter, we describe how the decision-making process at sports clubs differed along two dimensions (autocratic vs. democratic; fast vs. slow). Fig. 1.

### Motivating factors

We identified four motivating factors for sports clubs to start the decision-making process: 1) second-hand smoke as a problem, 2) intolerance of smoking behavior, 3) advantages of an outdoor SFP, and 4) external pressure to become smoke-free.

#### Second-hand smoke as a problem

At the majority of sports clubs, people were increasingly complaining or making comments about SHS. According to respondents, people were annoyed by spectators who were smoking next to the field. This included parents lighting a cigarette while watching their children play. Smoking by spectators was experienced as bothersome mainly because it resulted in the involuntary inhalation of tobacco smoke by those on the field. Moreover, respondents mentioned that people were also inhaling SHS at places where smokers used to gather, such as near the entrance of the sports club.

“When we were not smoke-free yet, people were smoking during competitions. They were smoking next to the dugout, even during youth matches. People were making some comments about it, which made us think.” (Respondent 20, korfball).

#### Intolerance of smoking behavior

Smoking at the sports club was less and less tolerated according to most respondents. They explained that the social norm with regard to smoking had changed. Smoking was perceived as outdated and the adoption of an outdoor SFP was in line with the societal trend of creating smoke-free environments. Furthermore, respondents felt that smoking and sports (clubs) do not fit together. This attitude was commonly based on the observation that sports are healthy, while smoking is unhealthy.

#### Advantages of an outdoor SFP

According to respondents, the adoption of an outdoor SFP may have a number of advantages. First, it may positively alter the way people perceive the sports club, i.e., as a sporty and healthy environment. Second, it may attract new club members. For example, parents on the lookout for a sports club for their children may prefer clubs that are smoke-free. Finally, it may have a positive effect on potential smoking behavior, as it decreases the visibility of smoking and the possibilities to smoke. Respondents believed that it is of particular importance to set a good example for children and to discourage them from starting to smoke themselves.

“If we want to prevent the new generation from smoking, we have to set a good example. If children leave the tennis court and see adults smoking after a match, they will think: that is normal. First playing tennis, and then smoking a cigarette. It is about children not thinking that smoking and sports fit together.” (Respondent 14, tennis).

Regardless of the above-mentioned advantages of becoming smoke-free, respondents acknowledged that the adoption of an outdoor SFP might have some disadvantages as well. According to respondents, it may result in the loss of smoking club members, including important volunteers. Furthermore, difficulties may arise about the enforcement of the SFP, as club members may not want to act like “police officers”. Finally, being voluntary organizations with social functions, it is important for sports clubs to not exclude smokers but to make them feel welcome at the sports club as well.

#### External pressure to become smoke-free

A number of respondents reported that the board of the sports club experienced a certain external pressure to adopt an outdoor SFP. First, respondents noted that an increasing number of sports clubs in their direct environment had become smoke-free, which resulted in a feeling of embarrassment and “not wanting to stay behind”. Second, the adoption of an outdoor SFP was promoted by external organizations, such as municipalities, sports federations, and public health services. These organizations sent general information about an outdoor SFP or encouraged sports clubs to become smoke-free. According to a number of respondents, it was mainly municipalities which had a strong ‘voice’ and pushed sports clubs towards becoming smoke-free.

“I don’t think the municipality would have forced us. However, they did push it a lot. They also delivered those smoke-free signs, saying: “When you are smoke-free, you need to hang these up.” It was all highly motivating.” (Respondent 16, korfball).

### Participants

According to the respondents, the decision-making process involved different participants, each with their own specific role and function.

#### The board

The board was a major participant in the decision-making process. Sometimes, the decision to adopt an outdoor SFP was solely the responsibility of the board, without any involvement of other participants. At the majority of sports clubs, however, the board was responsible for managing a decision-making process that involved other participants as well, and bringing it to a conclusion. Respondents mentioned that the board took into consideration other people’s contributions before making the ‘final decision’.

#### Club members

At the majority of sports clubs, club members were – in one way or another – involved in the decision-making process. At some sports clubs, people were informally asked to give their opinion about the new policy. This included mostly smokers and “influential club members”. The latter included volunteers and members of certain club committees (i.e., the bar committee). At other sports clubs, a questionnaire was sent out to all club members to assess their support and preference with regard to the outdoor SFP and its comprehensiveness.

“We decided to send out a questionnaire, which all club members received. What were their wishes? Their ideas? Their preferences? Based on the outcomes of that questionnaire, we decided at the general membership meeting to become a smoke-free sports club, with a smoking area around the corner.” (Respondent 19, tennis).

Furthermore, the adoption of an outdoor SFP was often discussed during the sports club’s general membership meeting. At most sports clubs, club members could respond to the proposal of the board to become smoke-free. Less often, the adoption of an outdoor SFP was voted upon.

#### Working groups

Sometimes, a so-called ‘working group’ concerned itself with the decision-making process. These groups were formed in consultation with the board and consisted of influential club members. Smokers were often included in working groups as well.

“We formed a working group, including one board member, one hardcore smoker, one normal smoker, and two people who wanted smoking to be stopped. And with the five of us, we had to make some kind of policy proposal.” (Respondent 3, football).

#### Smokers

According to a number of respondents, smokers were significant participants in the decision-making process. They expressed reservations and were often critical of change. Consequently, smokers were able to delay the decision-making process or persuade the board to adopt a partial instead of a total outdoor SFP.

### Choice opportunities

At the majority of sports club, decisions were sought and discussed during both formal and informal choice opportunities. However, it was only during formal choice opportunities that decisions were reached.

#### Formal choice opportunities

Formal choice opportunities included 1) formal meetings of the board, 2) formal meetings of the working group, and 3) the sports club’s general membership meeting. The first two meetings took place on a regular basis (e.g., monthly), while the general membership meeting took place only once or twice a year. Respondents expressed that general membership meetings are frequently only visited by influential club members. Finally, at some sports clubs, the board had put the adoption of an outdoor SFP on a so-called ‘action list’, meaning that it was something they wanted to accomplish within a defined term.

“Our general membership meeting takes place in May. Which is actually the highest decision-making body within our organization. In real life, however, you shouldn’t expect too much from it. Mostly, it is only 30 to 40 influential club members who attend such meetings.” (Respondent 6, football).

#### Informal choice opportunities

Informal choice opportunities included informal consultations between the board, the working group, or other club members. To illustrate, respondents mentioned that people had been discussing the adoption of an outdoor SFP next to the field, or that board members had been approaching club members on the terrace or in the canteen to ask their opinion about the policy.

#### Lower priority

At some clubs, decisions regarding an outdoor SFP were postponed. According to respondents, sports clubs had to deal with more important issues such as financial problems or – especially in the past two years – the COVID-19 pandemic.

“Sometimes, as a board, you need to make decisions and you can’t accomplish everything you want at the same time.” (Respondent 6, football).

However, as soon as these issues were taken care of, there was time again for the board to start the decision-making process in relation to the adoption of an outdoor SFP.

### Solutions

#### Type of outdoor SFP

Sports clubs adopted either a total or a partial outdoor SFP. At sports clubs with a total outdoor SFP, smoking at the venue was not allowed anywhere or at any time. A partial outdoor SFP took various forms. A number of sports clubs provided a designated smoking area on or just outside their venue. Other clubs prohibited smoking only on specific days of the week (e.g., on Saturday) or during specific time slots (e.g., during the day). According to respondents, there were several reasons to adopt a partial instead of total outdoor SFP. First, the change from smoking being allowed to a total outdoor SFP was sometimes considered too big, and sports clubs wanted to give people the chance to get used to new smoking rules. Second, sports clubs wanted to concede to smokers and avoid alienation of club members who smoked. Finally, a number of sports clubs were afraid that as a result of a total outdoor SFP, people would gather at the entrance to smoke, which may have resulted in smoking being highly visible.

“The board was afraid that smokers would shift to the entrance. Consequently, when passing by, you would walk through a ‘hedge of smoke’, and it would be the first thing you see. So that was one of the reasons to provide a smoking area instead of implementing a total outdoor SFP.” (Respondent 7, field hockey).

#### Smoke-free in phases

A number of respondents mentioned that their sports club became smoke-free in phases. That is, the sports club began with the adoption of a partial outdoor SFP and, after some period, expanded this to a total outdoor SFP. The main reason for this was to let club members – especially smokers – gradually get used to an outdoor SFP and mentally prepare them for the next step: a total outdoor SFP.

#### Two dimensions of the decision-making process

In addition to the four streams of the GCM, further analysis of the results revealed that sports clubs followed different strategies with regard to the decision-making process, which we classified along two dimensions: 1) autocratic vs. democratic and 2) fast vs. slow decision-making.

#### Autocratic vs. democratic decision-making

This dimension refers to the extent to which club members were involved in the decision-making process. At some sports clubs, the decision-making process appeared to follow an autocratic strategy, in which the decision to adopt an outdoor SFP was made solely by the board. According to respondents, this strategy was chosen for two reasons: 1) involving other participants would possibly delay and/or complicate the decision-making process, and 2) the board members felt they were authorized to make the decision on their own.

“We didn’t ask permission at the general membership meeting. We thought that, as a board, we were allowed to decide that ourselves.” (Respondent 8, tennis).

At other sports clubs, the decision-making process followed a more democratic strategy. In those cases, involvement of club members was perceived as essential to increase support for the outdoor SFP and to strengthen the ‘final decision’ of the board. Within this democratic strategy, the level of involvement of club members varied between sports clubs. At the lowest level, the board approached some club members to ask their opinion about adopting an outdoor SFP. This concerned mainly smokers and influential club members, as the board wanted to make sure that they supported the new policy. At the highest level, the decision to adopt an outdoor SFP was accomplished by vote or based on broad consensus during the general membership meeting.

“At one point, we addressed a number of smokers personally to ask them questions like: What is your view on this? Are you willing to cooperate? Do you understand our problem?” (Respondent 15, korfball).

#### Fast vs. slow decision-making

This dimension refers to the speed of the decision-making process. At some sports clubs, the decision-making process was perceived as fast, while at other clubs it went more slowly. A number of factors appeared to accelerate the decision-making process: 1) an autocratic strategy of the board, 2) a low number of smoking club members, 3) broad consensus among club members about the importance of an outdoor SFP, and 4) the composition of the board. With regard to the latter, boards that did not contain smokers, were decisive, and were able to use momentum demonstrated a higher pace of decision-making in this matter.

“The adoption went very smoothly. We didn’t have to do a lot to convince people to stop allowing smoking.” (Respondent 19, korfball).

## Discussion

### Key findings

The aim of this study was to explore how sports clubs decide to adopt an outdoor SFP. We found that sports clubs had several motivating factors to start the decision-making process, including SHS being perceived as a problem, increasing intolerance of smoking behavior, perceived benefits of an outdoor SFP, and external pressure to become smoke-free. Although decision-making involved a variety of participants, board members, influential club members, and smokers usually played major roles. Decisions were sought and discussed during both formal and informal choice opportunities, but only finally made during formal choice opportunities. Sports clubs adopted either a partial or a total outdoor SFP, with some clubs taking on a step-by-step approach. Lastly, we found that sports clubs differed both in the extent to which club members were involved in the decision-making process (autocratic vs. democratic) and the speed of the decision-making process (fast vs. slow).

### Interpretation of findings

The results of this study reveal a growing intolerance of smoking behavior at sports clubs, which has been reported in recent studies at sports clubs as well [12, 21]. This strong non-smoking norm may be a result of the collective and coordinated efforts of a global community dedicated to tobacco control. Since the adoption of the WHO Framework Convention on Tobacco Control (FCTC), the number of countries adopting tobacco control measures (e.g., raising prices through taxation and mandating plain packaging of tobacco products) continues to rise year-on-year [6]. This potentially resulted in the de-normalization of smoking among societies and is likely to be reflected in the factors that we found to motivate sports clubs to start the decision-making process (e.g., intolerance of smoking behavior). In the current study, this non-smoking norm appeared to be strong enough to become smoke-free, despite possible disadvantages of an outdoor SFP (e.g., the loss of smoking club members).

Sports clubs sometimes experienced an external pressure to adopt an outdoor SFP due to an increasing number of smoke-free clubs in their direct environment. This finding is in line with a Dutch study that found that sports clubs have a higher chance to adopt an outdoor SFP when being surrounded by sports clubs that are already smoke-free [11]. We also found that some sports clubs were triggered to adopt an outdoor SFP by external organizations. Although this finding suggests that there is a key role for organizations outside the sports club, it does not support previous studies which have shown that sports clubs can react defensively when gaining the impression that something is being imposed on them from outside [22, 23]. It is possible that sports clubs accept the interference of external organizations if this interference consists of promoting instead of imposing an outdoor SFP.

We found that the board played a major role in the decision-making process at sports clubs according to respondents. Similar findings have been reported earlier [14, 24, 25], suggesting that characteristics of board members are important predictors of how decisions are made. Indeed, the results of the current study indicated that the speed of the decision-making process was linked among others to whether boards contained smokers, were decisive, and used momentum. Ideally, such characteristics are taken into account when selecting new board members. However, recruiting volunteers if often already challenging for sports clubs, especially for more demanding functions such as a board member [14].

Smokers were seen as important participants in the decision-making process as well. The board sometimes chose a partial outdoor SFP over a total outdoor SFP because they were afraid to marginalize or even loose smoking club members. Such fear may however not come true. A study among sports clubs that already implemented an outdoor SFP showed that after implementation, the majority of smokers supported the policy [21]. Such experiences of sports clubs that are already smoke-free may be used as “success stories” to show to other clubs that implementation of an outdoor SFP is feasible.

The majority of sports clubs in our sample opted for some kind of democratic decision-making strategy. However, at odds with the view of sports clubs as being democratic organizations [15–17], an autocratic strategy was applied at a few clubs as well. One might argue that autocratic decision-making is preferred in order to increase the number of sports clubs with an outdoor SFP rapidly. However, this strategy may be less suitable for sports clubs with many smokers or a lack of consensus about the importance of an outdoor SFP. At those sports clubs, it may be better to involve club members in the decision-making process in order to increasing support for the new policy.

Lastly, we found that the adoption of an outdoor SFP is not always considered to be a top priority by the board, as it may have more pressing issues on its agenda. It has been suggested that perceptions of the importance and urgency of an innovation is crucial for adopting it within an organization [26, 27]. Since sports clubs are driven by the voluntary effort of club members, choices need to be made regarding which issues to address first. This may be among the reasons why the majority of sports clubs in the Netherlands are not yet smoke-free, and this highlights the importance of emphasizing sports club’s incentives to start the decision-making process.

### Potential limitations

A number of limitations need to be noted regarding the present study. First, we asked respondents to share their experiences regarding an event that took place one to three years ago. Consequently, some people may have struggled to remember what happened, especially when the sports club became smoke-free in 2018 or 2019. Second, we interviewed just one representative per sports club. Triangulating perspectives would have increased the trustworthiness of the results. Third, the current study included only one smoker (5% of the study population). Since we found that smokers may play a major role in the decision-making process, future studies should include more smokers in order to gain a better understanding of their perspectives. Finally, over the past years, a few sports clubs have professionalized to some extent, for example by relying partly on payed staff. At these sports clubs, the GCM may be less suitable to assess decision-making processes.

### Implications

The findings of this study have a number of practical implications for national and local governments, as well as for other external organizations that aim to stimulate sports clubs to become smoke-free. First of all, these organizations can play a substantial role in the adoption of outdoor SFPs, as sports clubs turned out to be susceptible to external pressure. This role can take various forms. For example, municipalities may organize meetings for board members of sports clubs to encourage them to become smoke-free. Such meetings could include a presentation in which the de-normalization is emphasized since this de-normalization appeared to be an important motivating factor for sports clubs to become smoke-free. Second, different governments and organizations may work together to increase the number of smoke-free sports clubs. To illustrate, in the Netherlands, the Dutch Heart Foundation keeps track of sports clubs with an SFP. Because of this, local governments have insight into which sports clubs are lagging behind, enabling them to pay extra attention to these clubs. Third, boards of sports clubs should be advised on how to deal with smoking club members, e.g., by explaining that including smokers in the decision-making process may increase support and compliance. Moreover, positive experiences of sports clubs that already implemented an SFP (so called “success stories”) may further encourage sports clubs to become smoke-free as well. As a final implication, tailored advice may be given to boards of sports clubs regarding the application of a democratic or more autocratic style of decision-making. The chosen style should depend among others on characteristics of the sports club, such as the anticipated level of support for an SFP.

## Conclusions

Our study revealed that a number of factors motivated sports clubs to start the decision-making process. These factors were mainly linked to a strong non-smoking norm. Frequently, decision-making involved only a few participants, with the board, influential club members, and smokers playing key roles. Governments and other external organizations may contribute to an increased adoption of SFPs at sports clubs in various was, including organizing gatherings. They may advise clubs on strategies of decision-making and how to involve smokers in this process.

## Data Availability

Data available on request.

## Appendix I

- 1. How would you describe the sports club?
- 2. Since when has the sports clubs been smoke-free? *(solutions)*
- 3. What rules does the sports club have for smoking on the venue? *(solutions)*
- 4. To what extent did people smoke at the sports clubs before the introduction of the smoke-free policy? *(motivating factors)*
- 5. To what extent was smoking at the sports club perceived as a problem by the members of the sports club? *(motivating factors)*
- 6. Can you tell me something about the sports club’s interest to become smoke-free? *(motivating factors)*
- 7. According to the sports club, what was the added value of an outdoor smoke-free policy? *(motivating factors)*
- 8. What were the reasons for the sports club to not become smoke-free? *(motivating factors)*
- 9. To what extent was an outdoor smoke-free policy promoted by persons/organizations outside the sports club? *(motivating factors, participants)*
- 10. Which people within the sports club were involved in the decision-making process? *(participants)*
- 10.a. What was the role of the board?
- 10.b. What was the role of other club members?
- 11. Which persons/organizations outside the sports club were involved in the decision-making process? *(participants)*
- 12. When were discussions about the outdoor smoke-free policy held? *(choice opportunities)*
- 12.a. Was this mainly in a formal or informal setting? Or both?
- 12.b. Was the adoption of an outdoor smoke-free policy put on an action list?
- 13. When did the sports club decide on the smoke-free policy? *(choice opportunities)*
- 14. Why was earlier not the right time to adopt an outdoor smoke-free policy? *(motivating factors)*
